# Oridonin attenuates low shear stress-induced endothelial cell dysfunction and oxidative stress by activating the nuclear factor erythroid 2-related factor 2 pathway

**DOI:** 10.1186/s12906-022-03658-2

**Published:** 2022-07-07

**Authors:** Zhipeng Chen, Heqian Liu, Xiaoqi Zhao, Subinur Mamateli, Cheng Liu, Lei Wang, Jing Yu, Yutong Liu, Jing Cai, Tong Qiao

**Affiliations:** 1 Department of Vascular Surgery, Affiliated Drum Tower Hospital, Medical School of Nanjing University, Nanjing, Jiangsu 210008 P.R. China; 2 Nanjing Drum Tower Hospital Clinical College of Xuzhou Medical University, Nanjing, Jiangsu 210008 P.R. China; 3 Department of Orthopedics, Nanjing Medical University Affiliated Wuxi Second Hospital, Wuxi, Jiangsu 214000 P.R. China; 4 Jiangsu Key Laboratory of Molecular Medicine, Medical School of Nanjing University, Nanjing, Jiangsu 210093 P.R. China

**Keywords:** Oridonin, Low shear stress, Atherosclerosis, Endothelial cell dysfunction, Oxidative stress, Zebrafish, Nuclear factor erythroid 2-related factor 2

## Abstract

**Background:**

Atherosclerosis (AS) is the primary cause of cardiovascular disease and the incidence is extremely common; however, there are currently few drugs that can effectively treat AS. Although oridonin has been widely used to treat inflammation and cancer for numerous years, to the best of our knowledge, its protective effect against AS has not been reported. Therefore, the present study aimed to investigate whether oridonin attenuated AS.

**Methods:**

By using text mining, chemometric and chemogenomic methods, oridonin was predicted to be a beneficial agent for the treatment of AS. A parallel flow chamber was used to establish a low shear stress (LSS)-induced endothelial cell (EC) dysfunction model. Briefly, ECs were exposed to 3 dyn/cm^2^ LSS for 30 min and subsequently treated with oridonin or transfected with a small interfering RNA (siRNA) targeting nuclear factor erythroid 2-related factor 2 (NRF2). Reactive oxygen species (ROS), superoxide dismutase (SOD), malondialdehyde (MDA), glutathione (GSH) and glutathione disulfide (GSSG) in EA.hy926 cells were analyzed to determine the level of oxidative stress. The nitric oxide (NO) levels and mRNA expression levels of endothelial NO synthase (eNOS), endothelin-1 (ET-1) and prostaglandin synthase (PGIS) in EA.hy926 cells were analyzed to determine EC dysfunction. Furthermore, the mRNA and protein expression levels of NRF2 were analyzed using reverse transcription-quantitative PCR and western blot. In addition, zebrafish were fed with a high-cholesterol diet to establish a zebrafish AS model, which was used to observe lipid accumulation and inflammation under a fluorescence microscope.

**Results:**

We found LSS led to oxidative stress and EC dysfunction; this was primarily indicated through the significantly decreased SOD and GSH content, the significantly increased MDA, GSSG and ROS content, the upregulated mRNA expression levels of ET-1, and the downregulated NO levels and mRNA expression levels of eNOS and PGIS in ECs. Notably, oridonin could improve LSS-induced oxidative stress and EC dysfunction, and the effects of oridonin were reversed by the transfection with NRF2 siRNA. Oridonin also attenuated lipid accumulation and neutrophil recruitment at the LSS regions in the zebrafish AS model.

**Conclusions:**

In conclusion, the results of the present study suggested that oridonin may ameliorate LSS-induced EC dysfunction and oxidative stress by activating NRF2, thereby attenuating AS.

**Supplementary Information:**

The online version contains supplementary material available at 10.1186/s12906-022-03658-2.

## Introduction

Atherosclerosis (AS) is characterized by endothelial dysfunction, inflammation, progressive lipid deposition and vessel stiffness, which is accompanied by potential complications such as myocardial infarction or stroke [1, 2]. Previous studies have demonstrated that atherosclerotic coronary artery disease is the main cause of cardiovascular disease mortality [3, 4]. Thus, it remains a priority to identify an effective therapeutic method or drug to attenuate or cure AS.

Recently, network pharmacology has been proposed as a novel discipline to investigate the complex interactions of compounds and biology. The potential targets of compounds are identified on a proteome-wide scale with computational ligand-based target prediction methods [5]. Multiple studies have confirmed that combining computational and experimental methods can effectively discover novel targets for drugs [6, 7].

Oridonin, the major active ingredient of the traditional Chinese medicinal herb *Rabdosia rubescens*, was proven to possess antitumor activity, and it has been used to treat liver, esophageal and pancreatic cancer in the clinic [8–11]. Previous studies have also reported that oridonin had anti-inflammatory effects and that it could inhibit the proliferation of endothelial cells (ECs), which are closely related to the pathogenesis of AS [12, 13]. However, to the best of our knowledge, its protective effect against AS has not been reported. In the present study, the molecular mechanisms underlying the multi-target effects of oridonin in AS were determined. Based on critical text mining, and chemometric and chemogenomic methods, it was hypothesized that the key targets of oridonin in AS may include adenylate cyclase type1 (ADCY1), ADCY2, nuclear factor erythroid 2-related factor 2 (NRF2) and nuclear receptor subfamily 0 group B member 1 (NROB1).

Although AS has numerous systemic risk factors, such as hypertension, obesity and smoking, it preferentially develops near branches and bends in blood vessels, where the ECs are exposed to oscillatory or low shear stress (LSS) [14]. Since this discovery, SS has been illustrated to serve an important role in the occurrence of AS [15]. LSS was discovered to induce oxidative stress and EC dysfunction, including reducing the levels of nitric oxide (NO) and upregulating endothelin-1 (ET-1) expression levels, which eventually lead to plaque formation. However, the mechanism for the conversion of mechanical signals to biological signals in ECs is only partially understood. A few detectors of LSS have been proposed, including mechanosensitive transcription factors (MSTFs), which may also be promising therapeutic targets for the treatment of endothelial dysfunction or AS. NRF2 is a MSTF that can sense the blood flow and regulate EC physiology [16]. Therefore, the present study selected NRF2, as a potential target of oridonin, for further investigations.

The current study used both a LSS-induced EC dysfunction model and a high-cholesterol diet (HCD)-induced zebrafish AS model to verify the target, which was identified previously. The findings revealed that oridonin ameliorated LSS-induced EC dysfunction by activating NRF2, thereby attenuating AS.

## Materials and methods

### Test compounds, chemicals and reagents

Oridonin (NSC-250682) was purchased from Selleck Chemicals. MTT reagent (11465007001) was purchased from Sigma-Aldrich; Merck KGaA. The other chemicals and reagents were of analytical grade.

### Target identification

To identify the targets of oridonin and determine compound-target interaction profiles, an in silico approach was applied as described in our previous study [17]; this approach integrated text mining with chemometric and chemogenomic methods. In the present study, text mining was first used to identify the targets of oridonin; this was performed using the Traditional Chinese Medicine Systems Pharmacology Database and Analysis Platform (https://tcmspw.com/tcmsp.php), a database comprised of systems pharmacology data for drug discovery related to herbal medicines [18]. Subsequently, the virtual chemical fingerprint similarity ensemble approach method (http://sea.bkslab.org) was used to predict potential targets of oridonin. Finally, the potential target proteins identified were further subjected to The Pharmacogenomics Knowledge Base (http://pharmgkb.org), and Comparative Toxicogenomics Database (http://ctdbase.org) to remove noise and errors. In addition, this enabled an accurate view of oridonin targets for cardiovascular diseases, including ADCY1, ADCY2, NRF2 and NROB1 (Table 1). It was previously reported that NRF2 served an important role in LSS-induced EC dysfunction and AS [16, 19], while another previous study discovered that oridonin improved inflammation and oxidative stress by activating NRF2 [20]. Therefore, NRF2 was selected for further investigations.

**Table 1:** The dock score of oridonin targets

Gene name	Description	Dock score
ADCY1	Adenylate cyclase type 1	8.9359
ADCY2	Adenylate cyclase type 2	15.5202
NRF2	Nuclear factor erythroid 2-related factor 2	11.2796
NROB1	Nuclear receptor subfamily O group B member 1	10.8533

### Human umbilical vein EC (HUVEC) culture and viability assay

The HUVEC cell line, EA.hy926, was obtained from the American Type Culture Collection. EA.hy926 cells were cultured in DMEM (Invitrogen, Thermo Fisher Scientific, Inc.) supplemented with 10% FBS, 100 U/ml penicillin and 100 μg/ml streptomycin, and maintained at 37 °C in a 5% CO_2_ humified incubator. Upon the EA.hy926 cells reaching the logarithmic growth phase, the cells were treated with 0.1% DMSO or various concentrations of oridonin (0, 25, 50, 100, 200 or 400 μg/ml) for 24 h. The cell viability was determined using an MTT assay, with ≥3 independent experiments performed in triplicate.

### LSS experiment

Upon EA.hy926 cells reaching the logarithmic growth phase, the cells were seeded onto a glass slide (30 × 50 mm) and treated with 0.1% DMSO, 100 μg/ml oridonin or 100 μg/ml oridonin combined with the transfection of 0.1 μmol/l small interfering RNA (siRNA) targeting NRF2 (A01004, GenePharma Shanghai) for 24 h. Following treatment for 24 h, the LSS test was performed. Briefly, a parallel flow chamber (81331, Shanghai Medical Equipment Works Co., Ltd), which consists of two stainless steel plates and a silicone gasket, was used in the present study. The glass slide with confluent cells was placed on the lower plate of the chamber and then subjected to LSS induced by continuous fluid flow. SS values were modulated by the flow through the chamber.

### EC dysfunction assay

The NO levels, and ET-1, endothelial NO synthase (eNOS) and PGIS mRNA expression levels in EA.hy926 cells were analyzed to determine EC dysfunction. Following the LSS experiment, EA.hy926 cells were incubated with 50 μM NO-specific fluorescent dye, 4-Amino-5-Methylamino-2′,7′-Difluorofluorescein diacetate (DAF-FM diacetate S0019, Beyotime Institute of Biotechnology). Following the incubation, the EA.hy926 cells were washed twice with PBS and then visualized and analyzed via fluorescence microscopy. The fluorescence intensity was semi-quantified from ≥3 random fields of view per slide from three different slides.

The mRNA expression levels of ET-1, eNOS and PGIS in EA.hy926 cells were analyzed using reverse transcription-quantitative PCR (RT-qPCR). Briefly, following the LSS experiment, total RNA was extracted from the cells using TRIzol® reagent (15596026, Invitrogen, Thermo Fisher Scientific, Inc.), according to the manufacturer’s protocol. Total RNA was reverse transcribed into cDNA using the PrimeScript™ RT Master mix (Perfect Real-Time) (RR064A/B, Takara Bio, Inc.), according to the manufacturer’s protocol. The resultant cDNA was used as a template for qPCR analysis in the Thermal Cycler Dice® Real-Time system (Takara Bio, Inc.). The primers used for the qPCR were designed by Primer3 software and are listed in Table 2. The mRNA expression data are expressed as the relative expression ratio normalized to GAPDH.

**Table 2:** The primers used for real-time PCR

Gene	Species		Primer sequence (5′ → 3′)
ET-1	Homo	Forward	GGCTGAAGGATCGCTTTGAGA
		Reverse	GCTCAGCGCCTAAGACTGTTT
eNOS	Homo	Forward	CTGGCTACAAGCACCGTGA
		Reverse	GGTTTCCAGCCCTGCTGTAT
PGIS	Homo	Forward	ATTACAACATGCCCTGGGGG
		Reverse	TGCGTTGATCAGCTCCAAGT
NRF2	Homo	Forward	AGGTTGCCCACATTCCCAAA
		Reverse	ACGTAGCCGAAGAAACCTCA
HMOX1	Homo	Forward	TAGAAGAGGCCAAGACTGCG
		Reverse	GGGCAGAATCTTGCACTTTGTT
NQO1	Homo	Forward	GGTTTGGAGTCCCTGCCATT
		Reverse	GCCTTCTTACTCCGGAAGGG
GAPDH	Homo	Forward	CCATGGGGAAGGTGAAGGTC
		Reverse	GCGCCCAATACGACCAAATC

### Oxidative stress assay

Following the LSS experiment, EA.hy926 cells were collected to measure secreted superoxide dismutase (SOD) activity, malondialdehyde (MDA) content, glutathione (GSH) content and glutathione disulfide (GSSG) content using ELISAs (S8530, BC0025, BC1170, BC1185, Beijing Solarbio Science & Technology Co., Ltd.), according to the manufacturers’ protocols.

Following the LSS experiment, EA.hy926 cells were also incubated with the ROS-specific fluorescent dye dihydroethidium (DHE S0063, 50 μM, Beyotime Institute of Biotechnology). Following the incubation, the EA.hy926 cells were washed in PBS twice and then visualized and analyzed via fluorescence microscopy. The fluorescence intensity was semi-quantified from ≥3 random fields of view per slide from three different slides.

### Western blot analysis

Following the LSS experiment, EA.hy926 cells were harvested for Western blot analysis. Briefly, the proteins were extracted using RIPA buffer (89900, Thermo Fisher Scientific, Waltham, UK). According to the manufacturer’s protocol, protein concentrations were determined by the BCA protein assay kit (P0012S, Beyotime Biotech, Shanghai, China), and equal amounts of proteins were resolved on the 10% SDS-PAGE gel followed by transferring to the PVDF membranes. After blocked with 5% skim milk for 90 min at room temperature, the blots were incubated at 4 °C overnight with primary antibodies against NRF2 (16396-1-AP, Proteintech Group, Inc., Chicago, IN, USA), HMOX1 (10701-1-AP, Proteintech Group, Inc.), eNOS (27120-1-AP, Proteintech Group, Inc.), SOD2 (ab171738, Abcam, Cambridge, MA, USA), and Beta Actin (ab8226, Abcam, Cambridge, MA, USA) as needed. After that, the membranes were further probed by the HRP-conjugated secondary antibodies. Blotted protein bands were visualized with enhanced chemiluminescence detection reagents (Thermo Fisher Scientific). Relative changes in protein expression were estimated from the mean pixel density using Image J, normalized to Beta Actin.

### Zebrafish AS model

Zebrafish lines used in this research were purchased from China Zebrafish Resource Center (CZRC, China). All studies involving zebrafish manipulations were approved by the institutional animal use and care committee of Nanjing Drum Tower Hospital. All animal experiments were performed in accordance with the National Institutes of Health Guidelines for the Care and Use of Laboratory Animals. About the euthanasia of zebrafish, we put zebrafish larvae in a 2 g/L tricaine solution for about 1 minute for euthanasia, meanwhile we use the lost of righting reflex, opercular movements and the silence of heart beating to confirm death. The accumulation of lipids in zebrafish blood vessels was detected to reflect early atherosclerotic plaque formation. As determined from a previous study [21], zebrafish larvae were fed with a HCD to establish a zebrafish AS model. In total, 5 days post-fertilization (dpf) *Tg*(*fli1:EGFP*) zebrafish larvae, constitutively expressing GFP in the ECs, were fed for 10 days with the HCD enriched with 4% cholesterol and supplemented with 10 μg/g fluorescent cholesteryl ester analog. A large amount of red fluorescent lipid accumulation in the zebrafish green blood vessels could be observed under a fluorescence microscope.

### Detection of the effect of oridonin treatment on early AS plaque formation

The 5 dpf *Tg*(*fli1:EGFP*) zebrafish larvae were randomly divided into five groups: i) Control group; ii) AS group; iii) 1 mg/l oridonin group; iv) 50 mg/l oridonin group; and v) 100 mg/l oridonin group. In the control group, the larvae were fed with 10 μg/g fluorescent cholesteryl ester analog for 10 days. In the AS group, the larvae were fed for 10 days with the HCD enriched with 4% cholesterol, and supplemented with 10 μg/g fluorescent cholesteryl ester analog. In the various concentrations of oridonin (1, 50 and 100 mg/l) treatment groups, the larvae were fed for 10 days with the HCD enriched with 4% cholesterol, and supplemented with 10 μg/g fluorescent cholesteryl ester analog. At the same time, the larvae were treated with various concentrations of oridonin (1, 50 and 100 mg/l). Images of the caudal vasculature in the live larvae revealed that the vasculature of the control and HCD larvae were stained diffusely red, with bright fluorescent lipid deposits in the blood vessel wall visible in the HCD larvae. A study previously reported that these accumulated lipids are similar to the plaques observed during early AS [22].

### Detecting inflammation in the zebrafish AS model

The presence of inflammation in *Tg(mpx:EGFP)* zebrafish was subsequently observed through specifically labeling the neutrophils with GFP. In the control group, 5 dpf *Tg(mpx:EGFP)* zebrafish larvae were fed with conventional feed for 10 days. In the AS group, 5 dpf *Tg(mpx:EGFP)* zebrafish larvae were fed for 10 days with a HCD enriched with 4% cholesterol. In the various concentrations of oridonin (1, 50 and 100 mg/l) treatment groups, 5dpf *Tg(mpx:EGFP)* zebrafish larvae were fed for 10 days with the HCD enriched with 4% cholesterol, and treated with various concentrations of oridonin (1, 50 and 100 mg/l). The quantity and recruitment of neutrophils was observed under a fluorescence microscope to determine the levels of inflammation in each group of zebrafish.

### Determining the effect of oridonin treatment on the lipid levels in the zebrafish AS model

The 5 dpf wild-type AB-line zebrafish larvae were randomly divided into five groups as said above. In the control group, zebrafish larvae were fed with normal basal feed (which did not contain 4% cholesterol) for 10 days. In the AS group, zebrafish larvae were fed a HCD enriched with 4% cholesterol for 10 days. In the various concentrations of oridonin (1, 50 and 100 mg/l) treatment groups, zebrafish larvae were fed for 10 days with an HCD enriched with 4% cholesterol and treated with various concentrations of oridonin (1, 50 and 100 mg/l). After 10 days of feeding and 24 h of fasting, Nile Red staining was used to detect the lipid levels in each group of zebrafish. The stock solution (1.25 mg/ml) of Nile Red (N-1142, Invitrogen, Thermo Fisher Scientific, Inc.) was prepared in acetone and stored in the dark at − 20 °C. For the staining of the zebrafish, the stock solution was diluted to 50 ng/ml in egg water and incubated for 15 min at 28 °C in the dark. The fishes were washed with distilled water 3 times and anesthetized with a few drops of tricaine solution (0.2 mg/ml, pH 7.0, Sigma-Aldrich, Merck KGaA). The zebrafish were subsequently mounted in 4% methylcellulose and the extent of Nile Red staining was imaged using an Olympus SZX16 microscope (Olympus Corporation), which was used for yellow fluorescent imaging.

### Detection of the effect of oridonin on the oxidative stress in the zebrafish AS model

The 5 dpf wild-type AB-line zebrafish larvae were randomly divided into five groups as said above. The description of each group of zebrafish is consistent with that described in the section describing the detection of the lipid levels in the zebrafish AS model. After 10 days of feeding and 24 h of fasting, dichloro-dihydro-fluorescein diacetate was used to detect the levels of ROS in each group of zebrafish.

### Biochemical measurements

The 5 dpf wild-type AB-line zebrafish larvae were randomly divided into five groups as said above. The description of each group of zebrafish is consistent with that described in the section describing the detection of the lipid levels in the zebrafish AS model. After 10 days of feeding and 24 h of fasting, 5 larvae from each larva in each group were randomly selected and sacrificed as one sample, and six samples were prepared for testing each index. Triglyceride (TG) levels, total cholesterol (TC) levels, SOD activity and MDA levels were measured using commercial assay kits (Jiancheng Bioengineering Institute, Nanjing, China, http://www.njjcbio.com/), according to the manufacturer’s protocols. The results of the aforementioned assays were quantified using a multifunctional microplate reader.

### Statistical analysis

Statistical analyses were performed using SPSS 22.0 software (IBM Corp.) and data are presented as the mean ± standard deviation. The normal distribution was checked with the Shapiro-Wilk test. Statistical differences between groups were determined using an ANOVA with Bonferroni adjustment for multiple comparisons. *P* < 0.05 was considered to indicate a statistically significant difference.

## Results

### Target screening

**Fig. 1:**
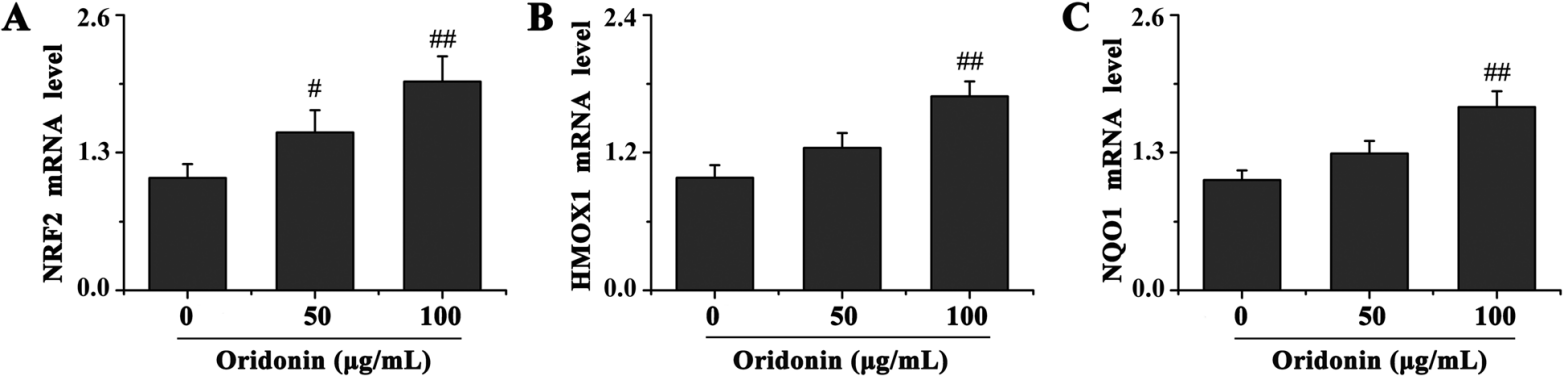
Oridonin upregulates the mRNA expression levels of (**A**) NRF2, (**B**) HMOX1 and (**C**) NQO1 in EA.hy926 cells. NRF2, nuclear factor erythroid 2-related factor 2; HMOX1, heme oxygenase 1; NQO1, NAD(P)H dehydrogenase [quinone] 1. The mRNA expressions have been normalized to Beta Actin, *n* = 3

Through combining text mining and chemogenomic prediction methods, 4 potential protein targets for oridonin were obtained (Table 1). It was previously discovered that NRF2 activation induced by high/laminar SS was important for EC adaptation to oxidative stress, in addition to exerting anti-inflammatory roles [23]. Furthermore, a previous study demonstrated that oridonin treatment improved inflammatory and oxidative stress by activating NRF2 [20]. The present study used RT-qPCR to determine the effect of oridonin treatment on the expression levels of NRF2 and its downstream target genes, heme oxygenase 1 (HMOX1) and NAD(P)H dehydrogenase [quinone] 1 (NQO1). The results revealed that oridonin treatment significantly upregulated the mRNA expression levels of NRF2, HMOX1 and NQO1 in EA.hy926 cells (Fig. 1A-C). Therefore, NRF2 was further investigated in the present study.

### Regulation of NRF2 in endothelial cells by LSS and oridonin

**Fig. 2:**
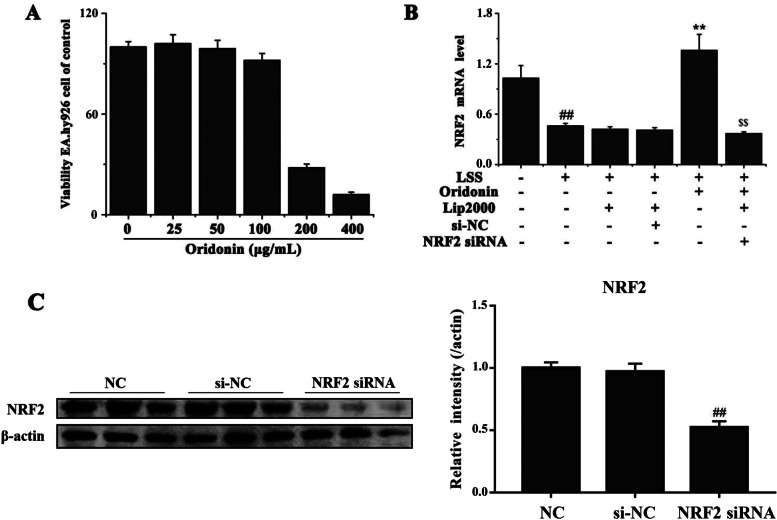
Endothelial cell viability assay and the confirmation of NRF2 siRNA transfection. **A** Effect of oridonin on EA.hy926 cell viability. EA.hy926 cells were treated with various concentrations of oridonin (0-400 μg/ml), and the viability was determined using an MTT assay at 24 h. **B** Genetic silencing of NRF2 counteracted the effects of oridonin treatment on the mRNA expression levels of NRF2. **C** The protein-level confirmation of NRF2 siRNA transfection

EA.hy926 cells were treated with various concentrations of oridonin (0, 25, 50, 100, 200 or 400 μg/ml) for 24 h, and the cell viability was determined using an MTT assay at 24 h. As shown in Fig. 2A, low doses of oridonin (0-100 μg/ml) did not inhibit cell proliferation, while higher doses of oridonin (200-400 μg/ml) inhibited cell proliferation. The half maximal inhibitory concentration value of oridonin was 337.84 μg/ml at 24 h. Based on these results, 100 μg/ml oridonin was used in further studies.

**Fig. 3:**
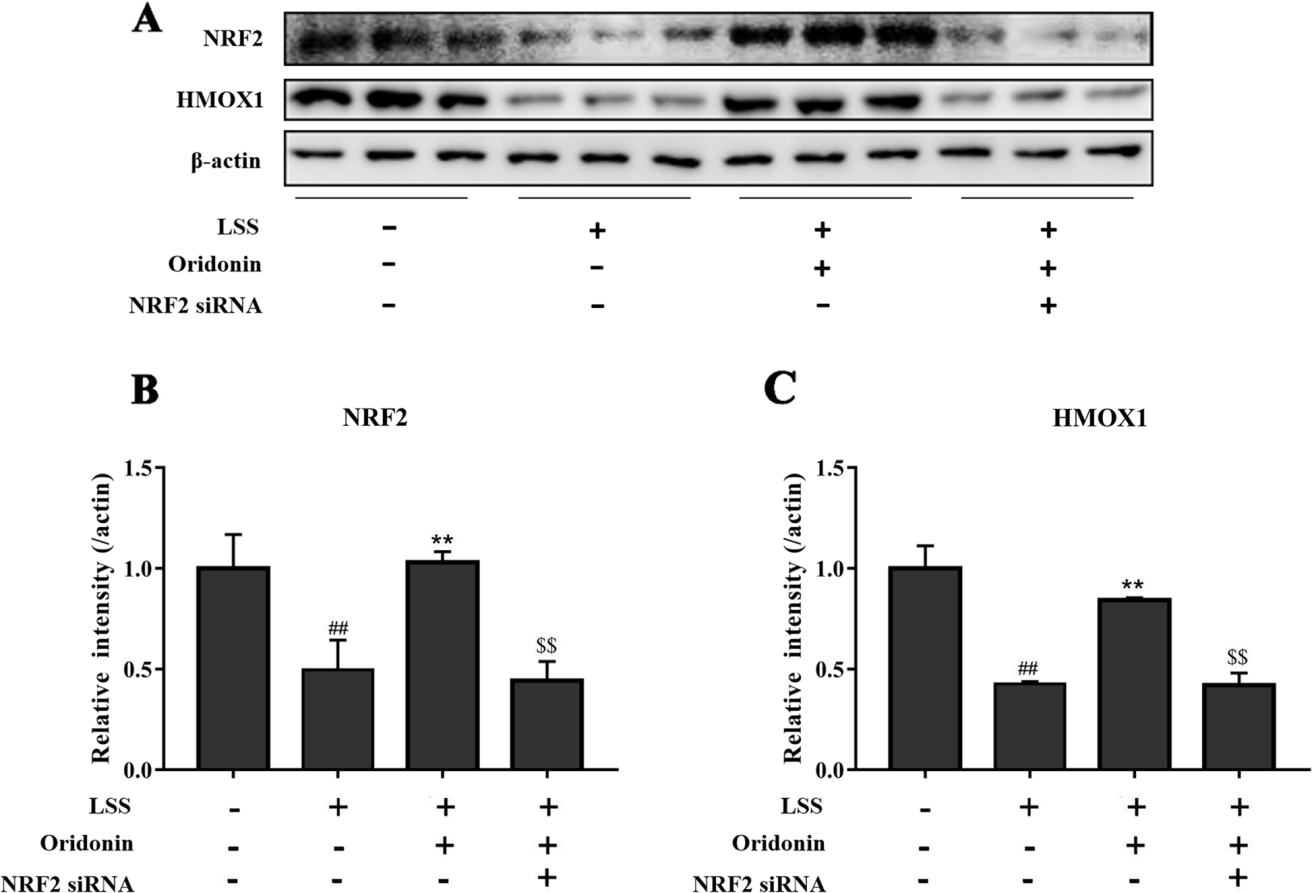
Regulation of NRF2 and HMOX1 in endothelial cells by LSS and oridonin. **A-C** Results of the western blot revealed that oridonin treatment counteracted the LSS-induced downregulation of NRF2 and HMOX1 by activating NRF2. ##*P* < 0.01 vs. LSS (−) + oridonin (−) + NRF2 siRNA (−); **P < 0.01 vs. LSS (+) + oridonin (−) + NRF2 siRNA (−); $$*P* < 0.01 vs. LSS (+) + oridonin (+) + NRF2 siRNA (−). LSS, low shear stress; NRF2, nuclear factor erythroid 2-related factor 2; HMOX1, heme oxygenase 1; siRNA, small interfering RNA

Similar to our previous study [24], EA.hy926 cells were exposed to laminar flow with a value of 0 or 3 dyn/cm^2^ for 30 min. A siRNA sequence targeting NRF2 was used to silence its expression, which was subsequently verified by RT-qPCR and western blot (Fig. 2B-C and Fig. S1). The mRNA level and protein expression of NRF2 were detected by RT-qPCR and western blot; as shown in Fig. 3A-C, LSS significantly reduced the expression of NRF2 and HMOX1 in endothelial cells, while the treatment with 100 μg/ml oridonin significantly increased the expression of NRF2 and HMOX1. Particularly, the genetic knockdown of NRF2 counteracted the effects of oridonin on the NRF2 and HMOX1 expression.

### Oridonin improves LSS-induced EC dysfunction by activating NRF2

**Fig. 4:**
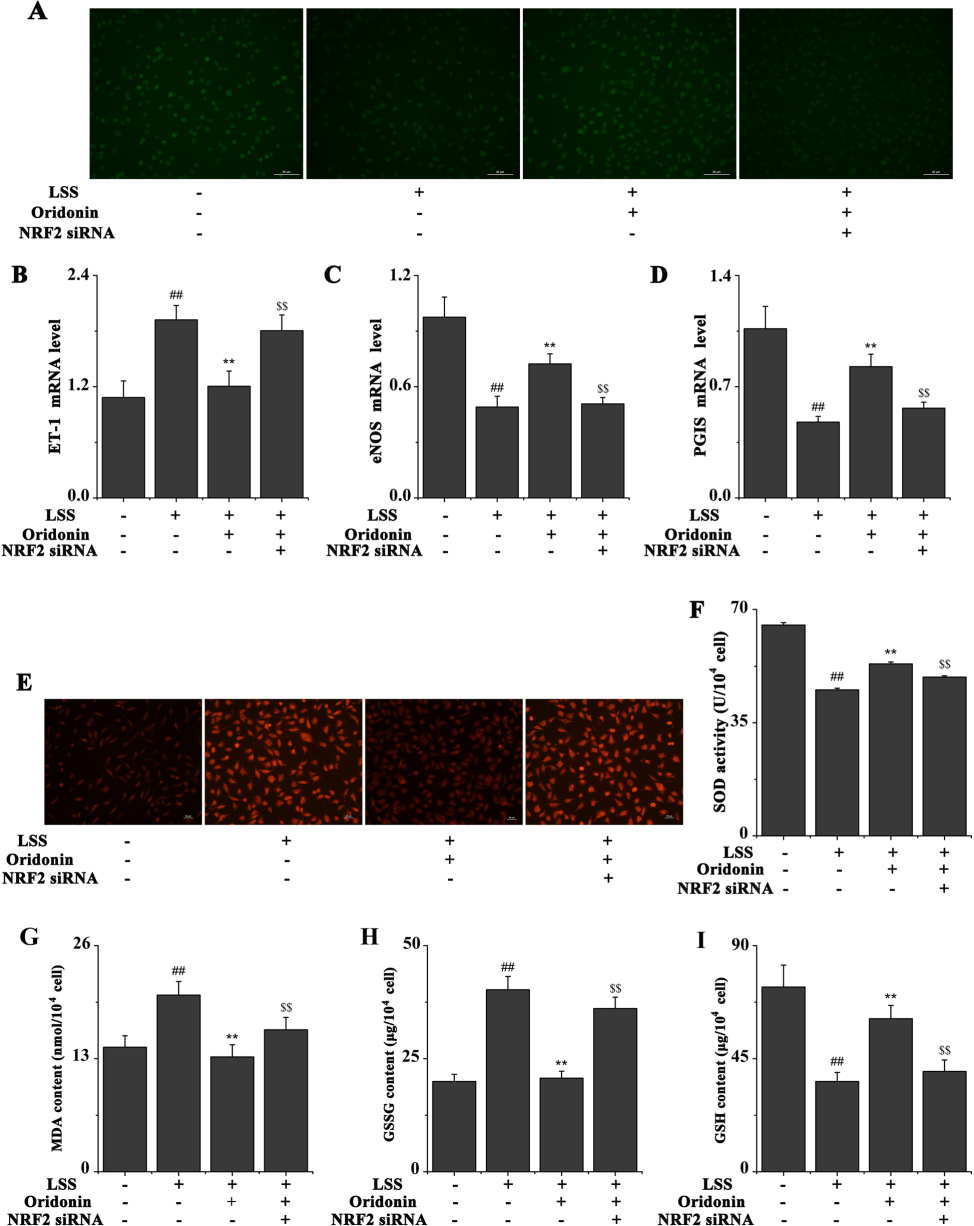
Oridonin improves LSS induced EC dysfunction and oxidative stress by activating NRF2. **A** Results of the fluorescent probe DAF-FM diacetate revealed that oridonin treatment increased the LSS-induced reduced NO activity by activating NRF2. **B** Oridonin treatment decreased the LSS-induced upregulation in ET-1 mRNA expression levels. **C** Oridonin treatment increased the LSS-induced downregulation in eNOS mRNA expression levels. **D** Oridonin treatment increased the LSS-induced downregulation in PGIS mRNA expression levels. **E** Results of the fluorescent probe dye DHE illustrated that oridonin treatment attenuated the LSS-induced increase in ROS activity in EA.hy926 cells. **F** Oridonin treatment increased the LSS-induced decrease in SOD activity in EA.hy926 cells. **G** Oridonin treatment decreased the LSS-induced increase in MDA content in EA.hy926 cells. **H** Oridonin treatment decreased the LSS-induced increase in GSSG content in EA.hy926 cells. **I** Oridonin treatment increased the LSS-induced decrease in GSH content in EA.hy926 cells. ^##^*P* < 0.01 vs. LSS (−) + oridonin (−) + NRF2 siRNA (−); ^**^P < 0.01 vs. LSS (+) + oridonin (−) + NRF2 siRNA (−); ^$$^*P* < 0.01 vs. LSS (+) + oridonin (+) + NRF2 siRNA (−). LSS, low shear stress; NRF2, nuclear factor erythroid 2-related factor 2; ET-1, endothelin-1; eNOS, endothelial nitric oxide synthase; PGIS, prostaglandin synthase; ROS, reactive oxygen species; SOD, superoxide dismutase; MDA, malondialdehyde; GSSG, glutathione disulfide; GSH, glutathione; siRNA, small interfering RNA

**Fig. 5:**
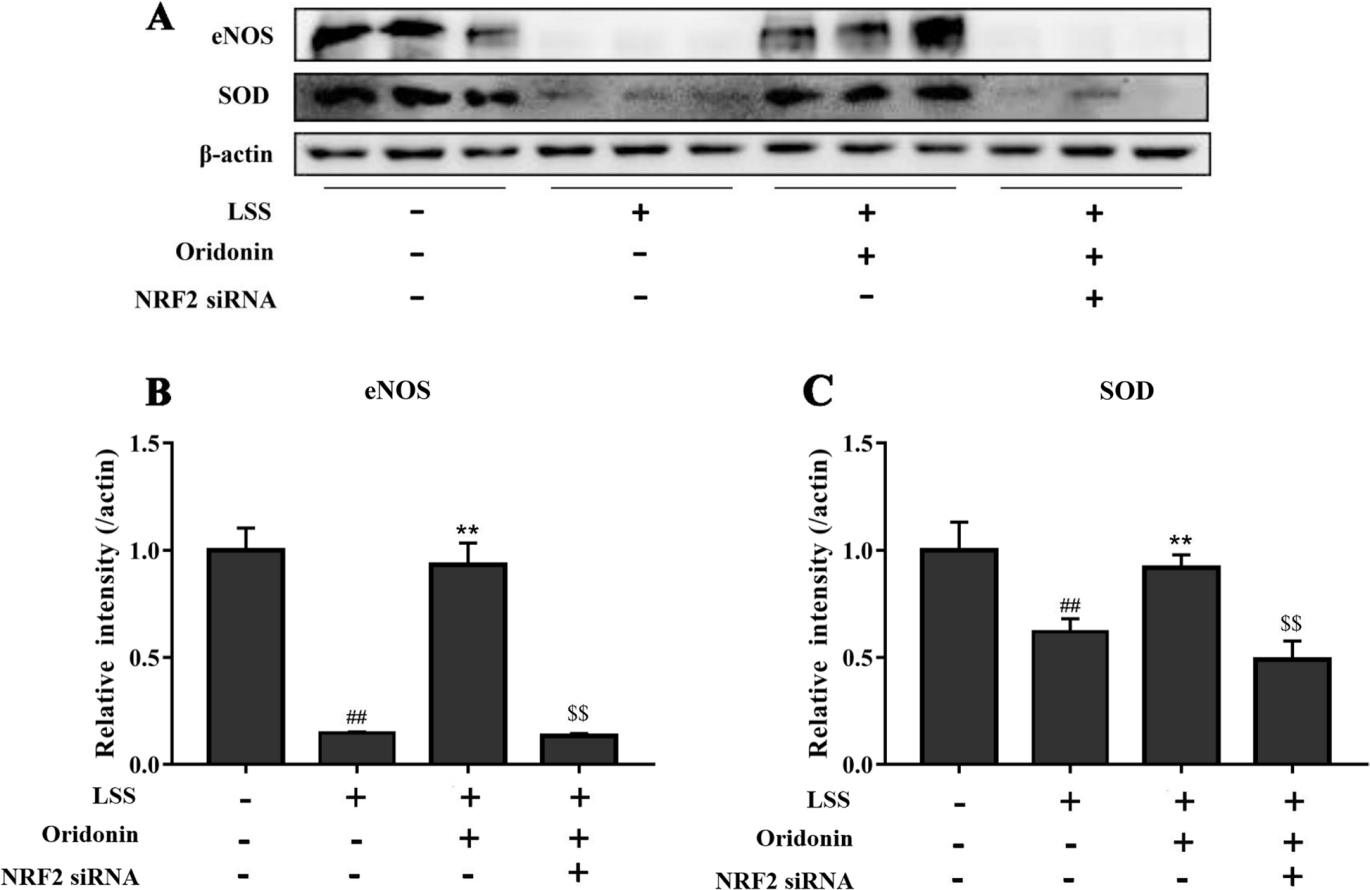
Oridonin improves LSS induced EC dysfunction and oxidative stress by activating NRF2. **A-C** Results of the western blot revealed that oridonin treatment counteracted the LSS-induced downregulation of eNOS and SOD by activating NRF2. ^##^*P* < 0.01 vs. LSS (−) + oridonin (−) + NRF2 siRNA (−); **P < 0.01 vs. LSS (+) + oridonin (−) + NRF2 siRNA (−); ^$$^*P* < 0.01 vs. LSS (+) + oridonin (+) + NRF2 siRNA (−). LSS, low shear stress; eNOS, endothelial nitric oxide synthase; SOD, superoxide dismutase; siRNA, small interfering RNA

The intracellular NO activity was analyzed using the fluorescent probe DAF-FM diacetate; as shown in Fig. 4A, LSS significantly reduced the NO activity, while the treatment with 100 μg/ml oridonin significantly increased NO activity. Notably, the genetic knockdown of NRF2 counteracted the effects of oridonin on the intracellular NO activity. The expression levels of EC dysfunction-related genes (ET-1, eNOS and PGIS) were also determined using RT-qPCR. As shown in Fig. 4B-D, LSS significantly downregulated the mRNA expression levels of eNOS and PGIS, while significantly upregulating the ET-1 mRNA expression levels. These changes in the expression levels were significantly inhibited by 100 μg/ml oridonin, whereas the genetic silencing of NRF2 counteracted the effects of oridonin treatment on the mRNA expression levels of ET-1, eNOS and PGIS. In addition, we chose eNOS for protein-level confirmation, and the similar results are shown in Fig. 5A-B.

### Oridonin improves LSS-induced oxidative stress by activating NRF2

The intracellular ROS activity was investigated using the fluorescent probe dye DHE. LSS significantly induced ROS activity, while the ROS activity was significantly inhibited by 100 μg/ml oridonin (Fig. 4E). Conversely, the genetic knockdown of NRF2 counteracted the effects of oridonin treatment on the activity of ROS. The levels of SOD, MDA, GSSG and GSH in EA.hy926 cells were also investigated. As shown in Fig. 4F-I, LSS significantly reduced SOD and GSH levels and significantly increased MDA and GSSG levels, while the effects of LSS were significantly inhibited by 100 μg/ml oridonin treatment. Meanwhile, the genetic silencing of NRF2 counteracted the effects of oridonin treatment on the levels of these oxidases. Also, we chose SOD for protein-level confirmation, and the similar results are shown in Fig. 5A and C.

### Oridonin reduces the formation of early plaques in a zebrafish AS model

**Fig. 6:**
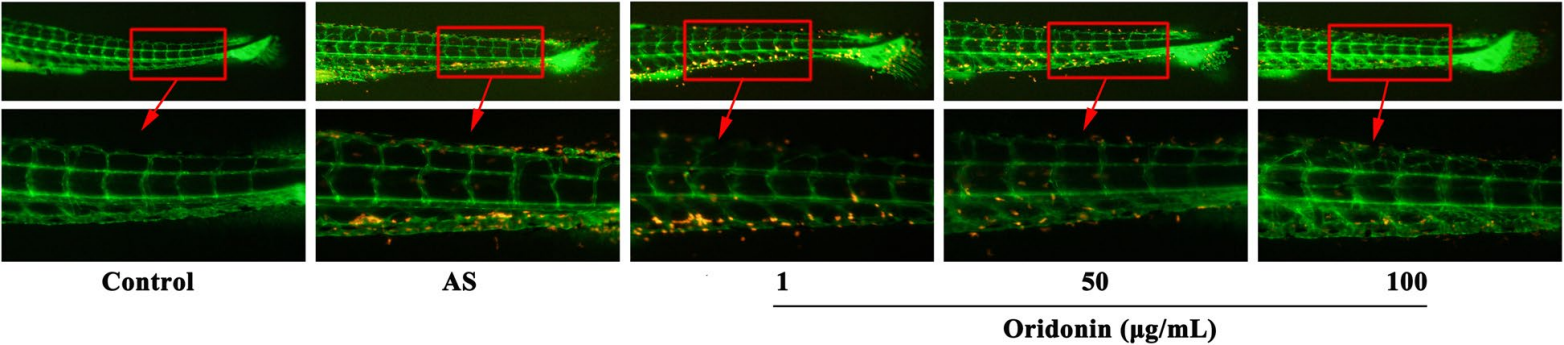
Oridonin reduces the formation of early plaques in AS zebrafish blood vessels. AS, atherosclerosis

Severe plaques formed in the blood vessels of the *Tg*(*fli1:EGFP*) zebrafish fed the HCD (Fig. 6), whereas the treatment with 50 and 100 μg/ml oridonin appeared to decrease the HCD-induced plaque formation. However, the treatment with 1 μg/ml oridonin had not significant effect on HCD-induced plaque formation.

### Oridonin reduces lipid accumulation in a zebrafish AS model

**Fig. 7:**
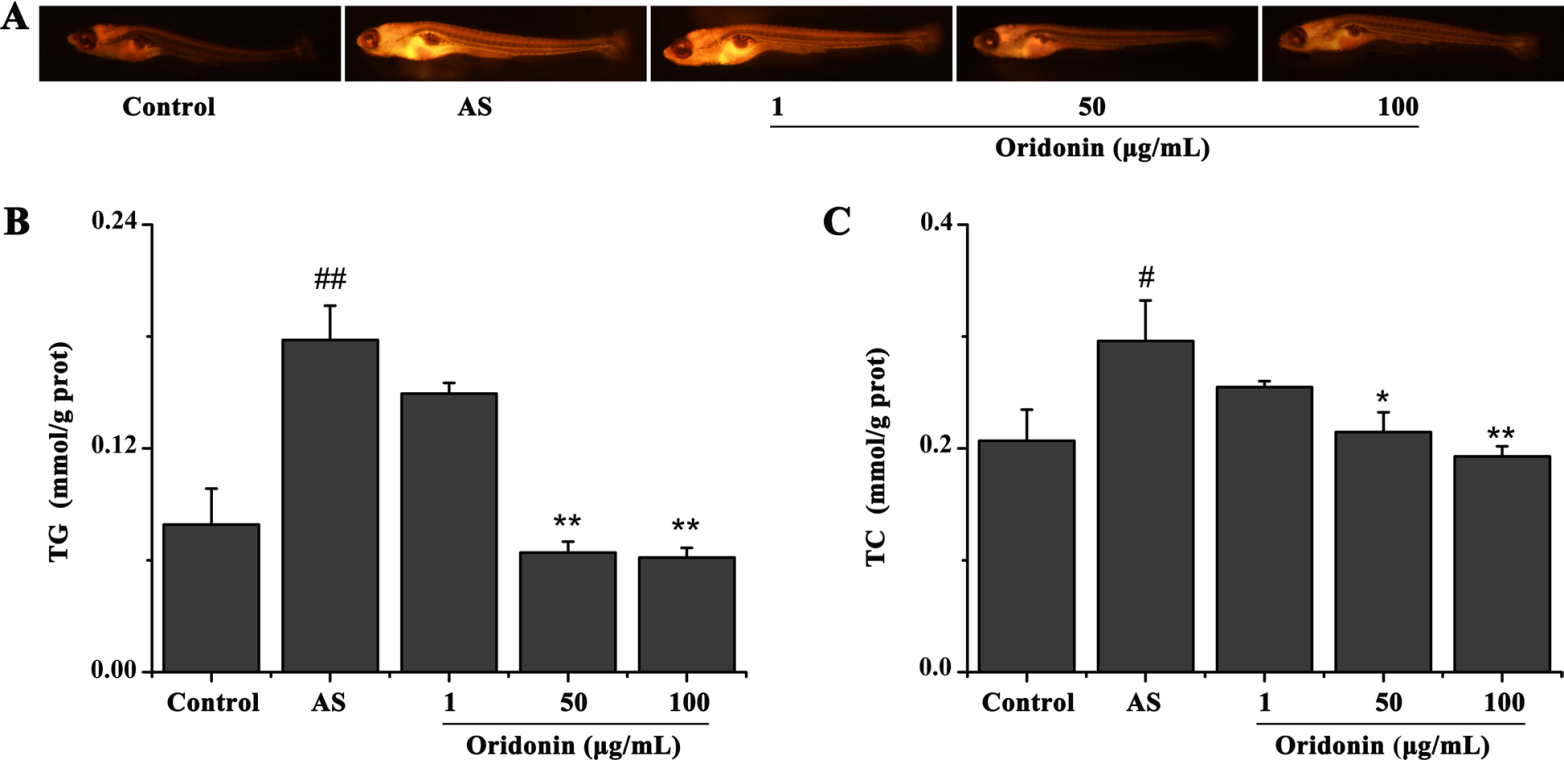
Oridonin reduces the lipid accumulation in a zebrafish AS model. **A** Results of the Nile Red staining revealed that oridonin treatment reduced the blood lipid levels in AS model zebrafish. Oridonin treatment reduced the (**B**) TG and (**C**) TC content in AS model zebrafish. ^#^*P* < 0.05, ^##^*P* < 0.01 vs. control group; ^*^P < 0.05, ^**^P < 0.01 vs. AS group. AS, atherosclerosis; TG, triglyceride; TC, total cholesterol

The lipid levels in zebrafish were detected using Nile Red staining. As shown in Fig. 7A, compared with the control group, the lipid levels were significantly increased in the AS group. However, compared with the AS group, the treatment with 50 or 100 μg/ml oridonin appeared to decrease the lipid levels, while 1 mg/l oridonin had no significant effect on the lipid levels. A similar result was also obtained by measuring the TG and TC contents in the zebrafish; compared with the control group, the levels of TGs and TC were significantly increased in the AS group (Fig. 7B and C). In contrast, compared with the AS group, the treatment with 50 or 100 μg/ml oridonin significantly decreased the levels of TGs and TC, while 1 mg/l oridonin did not markedly decrease the levels of TGs and TC.

### Oridonin reduces oxidative stress in a zebrafish AS model

**Fig. 8:**
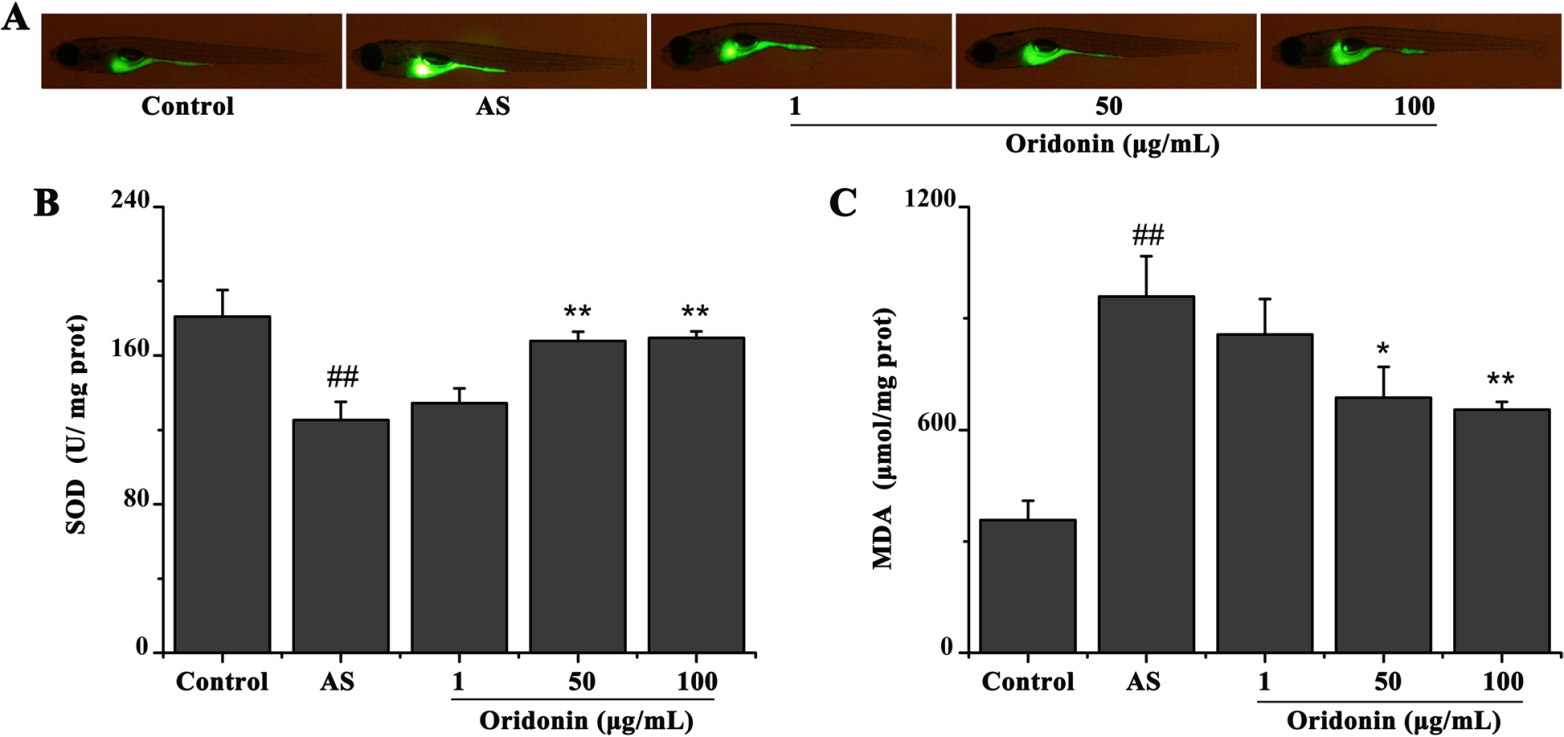
Oridonin reduces the levels of oxidative stress in a zebrafish AS model. **A** Results of the dichloro-dihydro-fluorescein diacetate assay revealed that oridonin treatment reduced the ROS activity in the zebrafish AS model. **B** Oridonin treatment increased the SOD activity in the zebrafish AS model. **C** Oridonin treatment reduced the MDA content in the zebrafish AS model. ^##^*P* < 0.01 vs. control group; ^*^P < 0.05, ^**^P < 0.01 vs. AS group. AS, atherosclerosis; ROS, reactive oxygen species; SOD, superoxide dismutase; MDA, malondialdehyde

As shown in Fig. 8A, compared with the control group, the ROS levels were significantly increased in the AS group. Conversely, compared with the AS group, the treatment with 50 or 100 μg/ml oridonin appeared to decrease the ROS levels, while 1 mg/l oridonin did not affect the ROS levels. Consistent results were obtained by measuring the SOD activity and MDA content in the zebrafish. That is, compared with the control group, the SOD activity was significantly decreased, while the MDA content was significantly increased, in the AS group (Fig. 8B and C). Meanwhile, compared with the AS group, the treatment with 50 and 100 μg/ml oridonin significantly reduced the MDA content and significantly increased SOD activity. Similar to the previous findings, 1 mg/l oridonin had no significant effect on the SOD activity and MDA content.

### Oridonin reduces inflammation in a zebrafish AS model

**Fig. 9:**
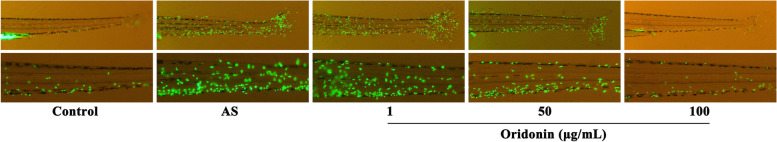
Oridonin reduces the number of neutrophils in a zebrafish atherosclerosis model

A large number of neutrophils were identified in the blood vessels of the *Tg(mpx:EGFP)* zebrafish fed the HCD, while the treatment with 50 or 100 μg/ml oridonin appeared to decrease the HCD-induced increase in neutrophils (Fig. 9). However, the treatment with 1 μg/ml oridonin did not significantly inhibit the HCD-induced increase in the number of neutrophils in the blood vessels.

## Discussion

The present study used a novel systems pharmacology method to identify new drugs for the potential treatment of cardiovascular diseases. The hubs and the centric elements of the network were analyzed to identify key targets, and through this analysis, 4 targets with high degrees were identified, namely ADCY1, ADCY2, NRF2 and NROB1. Out of these targets, NRF2 was selected for further investigations and it was discovered that oridonin treatment significantly upregulated the mRNA expression levels of NRF2, and its target genes, such as HMOX1 and NQO1, in EA.hy926 cells. In addition, the atheroprotective effects of oridonin on ECs were weakened following the genetic silencing of NRF2.

LSS has been reported to serve an important role in the occurrence of EC dysfunction and AS [25]. ECs, the innermost monolayer of the vessel wall, are the earliest cells to encounter SS; thus, they are the major cell type to sense the hemodynamic forces from the blood flow and transfer the mechanical signals to biochemical signals to regulate various signaling pathways. Therefore, improving EC dysfunction induced by LSS is hypothesized to be an effective way to prevent or cure AS. EC dysfunction is characterized by an imbalance between vasodilatory and vasoconstrictive molecules; these molecules, including prostacyclin (PGI_2_), NO and ET-1, are synthesized and released by ECs. PGI_2_ and NO are effective vasodilators and are produced by PGIS and eNOS, respectively, while ET-1 is a potent vasoconstrictor [26]. In the present study, a parallel flow chamber was used to establish an LSS-induced EC dysfunction in vitro model and it was subsequently discovered that LSS significantly reduced the activity of NO. In addition, LSS significantly downregulated the mRNA expression levels of eNOS and PGIS, while significantly upregulating ET-1 mRNA expression levels. More importantly, we reached similar conclusions through the western blot results of eNOS.

LSS was previously found to increase oxidative stress levels in ECs [24]. The findings of the present study illustrated that LSS significantly reduced the activity of SOD and GSH, while significantly increasing the levels of ROS, MDA, and GSSG. Notably, the oridonin treatment improved the LSS-induced oxidative stress, while the genetic silencing of NRF2 counteracted the effects of oridonin treatment on LSS-induced oxidative stress. These results suggested that oridonin may improve LSS-induced oxidative stress by activating NRF2. In addition, the results of *Tg(mpx:EGFP)* zebrafish indicate that HCD will increase the level of inflammation in zebrafish, and oridonin can effectively inhibit the increase in inflammation caused by HCD.

NRF2 is an important mechanosensitive transcription factor that regulates the expression of a group of genes responsible for inflammation and oxidative stress. Thus, it is considered to have a close relationship with EC dysfunction and AS. In the high/laminar SS regions of the vasculature, NRF2 was discovered to be activated, where it participated in the adaptative mechanism of the ECs to oxidative and inflammatory stress [16]. In the current study, the expression levels of NRF2 were downregulated by LSS. Thus, identifying a drug targeting the downregulation of NRF2 in the LSS regions may be the future of AS treatment.

To investigate whether oridonin treatment attenuated AS, zebrafish were fed a HCD to establish a zebrafish AS model. The zebrafish is a unique vertebrate model that combines the advantageous characteristics of invertebrate models (small size, powerful genetic tractability, high fecundity, ease of maintenance and relatively low costs) with a high degree of evolutionary conservation with mammals. Thus, zebrafish are invaluable, not only for studying vertebrate development and physiology but also for modeling human diseases [27]. Zebrafish are also used to study AS, in which the hypercholesterolemia zebrafish is currently the most commonly used zebrafish AS model [21]. In the present study, severe lipid accumulation occurred in the blood vessels of the zebrafish fed a HCD, and studies have previously shown that this accumulation of lipids in the blood vessels is similar to the early plaques of AS [22]. These results further suggested that HCD-fed zebrafish larvae may be used to study human AS. A recent study illustrated that the arteriovenous differentiation was incomplete during the larval stage of zebrafish, thus the veins and arteries are considered to be similar during this period. The only difference is that the blood flow in the veins is much slower, which signifies that the wall SS induced by the blood flow is lower in the veins, especially in those near the tail [28]. This hypothesis is consistent with the results obtained in the present study, as the lipid accumulation, which was represented by red fluorescence, was more severe in the veins near the tail. However, the results revealed that oridonin treatment could significantly reduce the accumulation of lipids in the blood vessels of a zebrafish AS model. These findings indicated that oridonin may exert a beneficial therapeutic effect on early atherosclerotic plaque formation caused by LSS.

It is generally considered that hyperlipidemia, inflammation and oxidative stress lead to the initiation and development of AS [29–31]. In the present study, a zebrafish AS model was established by feeding zebrafish with a HCD for 10 days. Zebrafish in the AS group demonstrated signs of hyperlipidemia, oxidative stress and inflammation, as expected. These results further illustrated that zebrafish can be used to study AS. Notably, oridonin treatment significantly improved the HCD-induced hyperlipemia, oxidative stress and inflammation. These findings also indicated that the anti-AS effect of oridonin may be related to its lipid-lowering, antioxidant and anti-inflammatory effects.

There are many limitations in this study, we revealed the potential of oridonin in the treatment of atherosclerosis, and briefly studied the role of NRF2 in the treatment of atherosclerosis. The mechanism of our research is not deep enough and we will conduct more research in subsequent experiments to specify how dose oridonin overcome shear stress-induced endothelial dysfunction and oxidative stress. A fluorescence quantification by flow cytometry will be a good supplementary result to show the changes between intracellular NO activity and we will supplement it in later experiments. Most importantly, we only found that oridonin can affect the expression of NRF2, but whether NRF2 is the target of oridonin needs more research in the future.

In conclusion, the findings of the present study revealed that oridonin exerted effects in LSS-induced EC dysfunction and HCD-induced hyperlipidemia, inflammation and oxidative stress, and inhibited plaque formation in AS as observed from a sequence of testing in-silico target identification, human umbilical culture, and a zebrafish model. Therefore, in the future, oridonin may be considered as a potential therapeutic option for AS.

## Supplementary Information

**Additional file 1: Figure S1.** Effects of NRF2 siRNA on NRF2 mRNA without any other treatment. ##*P* < 0.01 when compared with si-NC, NRF2, nuclear factor erythroid 2-related factor 2, siRNA, small interfering RNA.

**Additional file 2: Figure S2.** Original images of western blot in Fig. 2. NRF2, nuclear factor erythroid 2-related factor 2.

**Additional file 3: Figure S3.** Original images of western blot in Fig. 3. NRF2, nuclear factor erythroid 2-related factor 2, HMOX1, heme oxygenase 1.

**Additional file 4: Figure S4.** Original images of western blot in Fig. 5. eNOS, endothelial nitric oxide synthase SOD, superoxide dismutase.

## Data Availability

All data generated or analysed during this study are included in this published article and its supplementary information files.

## Abbreviations

AS: Atherosclerosis
EC: Endothelial cell
LSS: Low shear stress
SS: Shear stress
NRF2: Nuclear factor erythroid 2-related factor 2
NQO1: NAD(P)H:quinoneoxidoreductaseNQO1:EC1.6.99.2
HMOX1: Heme oxygenase
ADCY1: Adenylate cyclase 1
ADCY2: Adenylate cyclase 2
MTT: Thiazolyl blue
RT-qPCR: Realtime quantitative PCR
siRNA: Small interfering RNA
ROS: Reactive oxygen species
LDL: Low-density lipoprotein
ox-LDL: Oxidized Low-density lipoprotein
NF-κB: Nuclear factor kappa-B
HIF-1α: Hypoxia inducible factor-1-alpha
IL-6: Interleukin-6
TNF-α: Tumor necrosis factor-α
MSTFs: Mechanosensitive transcription factors
NADPH: Nicotinamide adenine dinucleotide phosphate
SOD: Superoxide dismutase
MDA: Malondialdehyde
GSH: Glutathione
GSSG: Glutathione (Oxidized)
CMC-Na: Carboxymethylcellulose sodium
HCD: High-cholesterol diet
DMSO: Dimethyl sulfoxide
DMEM: Dulbecco’s modified Eagle’s medium
DEPC: Diethy pyrocarbonate
MAPK: Mitogen-activated protein kinase
NO: Nitric oxide
PBS: Phosphate buffered saline
FBS: Fetal calf serum
SDS: Sodium dodecylsulphate
MCP-1: Monocyte chemotactic protein-1
ICAM-1: Intercellular cell adhesion molecule-1
VCAM-1: vascular cell adhesion molecule-1
vWF: Von willebrand factor
ET-1: Endothelin -1
PGIS: Prostacyclin synthase
PGI2: Prostaglandin I-2
MS-222: Tricaine
