# Supplementary figures and images for: Oridonin attenuates low shear stress-induced endothelial cell dysfunction and oxidative stress by activating the nuclear factor erythroid 2-related factor 2 pathway
Source: BMC Complement Med Ther. 2022 Jul 7;22:180. doi: 10.1186/s12906-022-03658-2 (PMC9261036; doi:10.1186/s12906-022-03658-2)

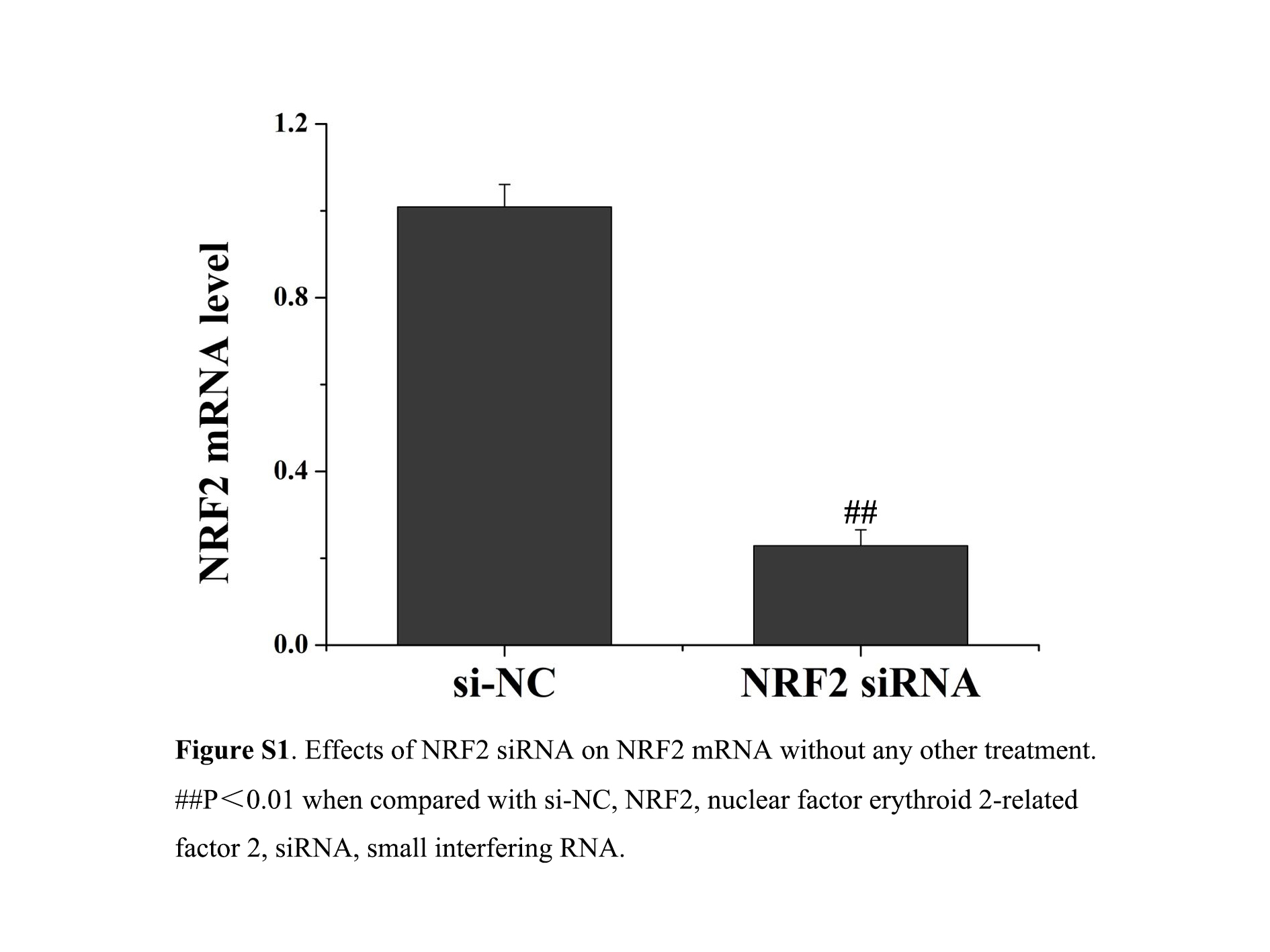

Supplement: Supplementary file 1 — Additional file 1: Figure S1. Effects of NRF2 siRNA on NRF2 mRNA without any other treatment. ##P < 0.01 when compared with si-NC, NRF2, nuclear factor erythroid 2-related factor 2, siRNA, small interfering RNA. [file 12906_2022_3658_MOESM1_ESM.jpg]

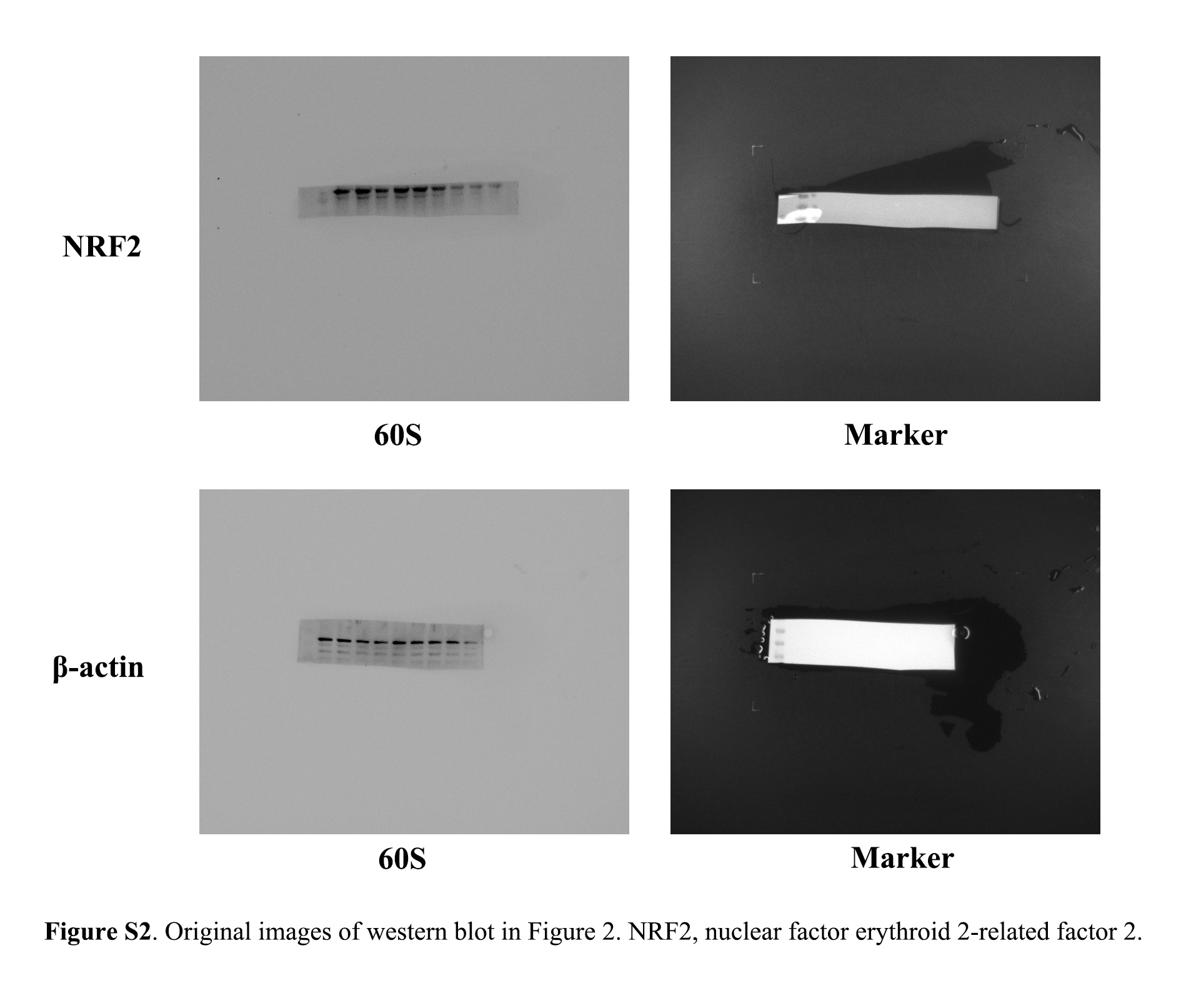

Supplement: Supplementary file 2 — Additional file 2: Figure S2. Original images of western blot in Fig. 2. NRF2, nuclear factor erythroid 2-related factor 2. [file 12906_2022_3658_MOESM2_ESM.jpg]

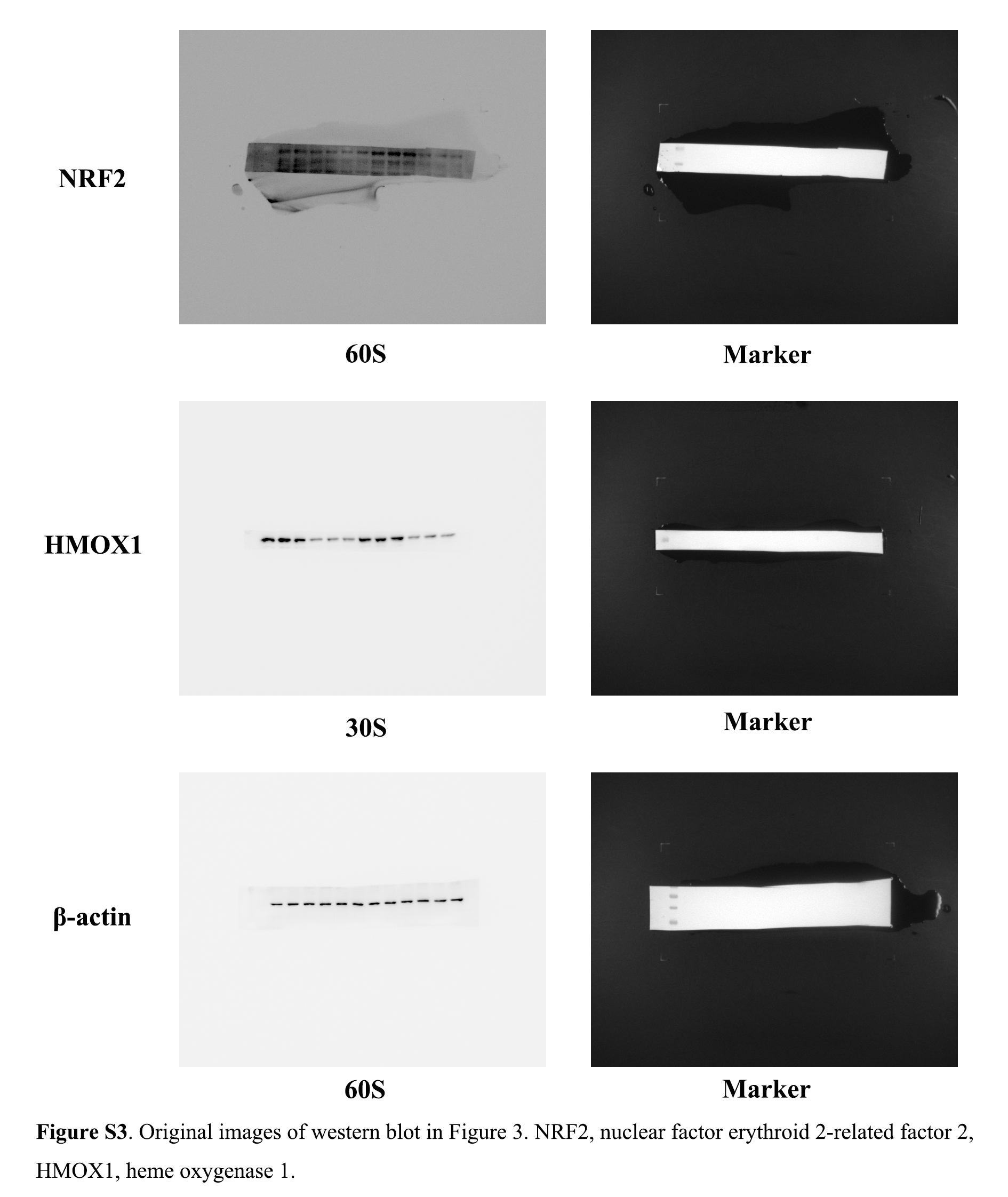

Supplement: Supplementary file 3 — Additional file 3: Figure S3. Original images of western blot in Fig. 3. NRF2, nuclear factor erythroid 2-related factor 2, HMOX1, heme oxygenase 1. [file 12906_2022_3658_MOESM3_ESM.jpg]

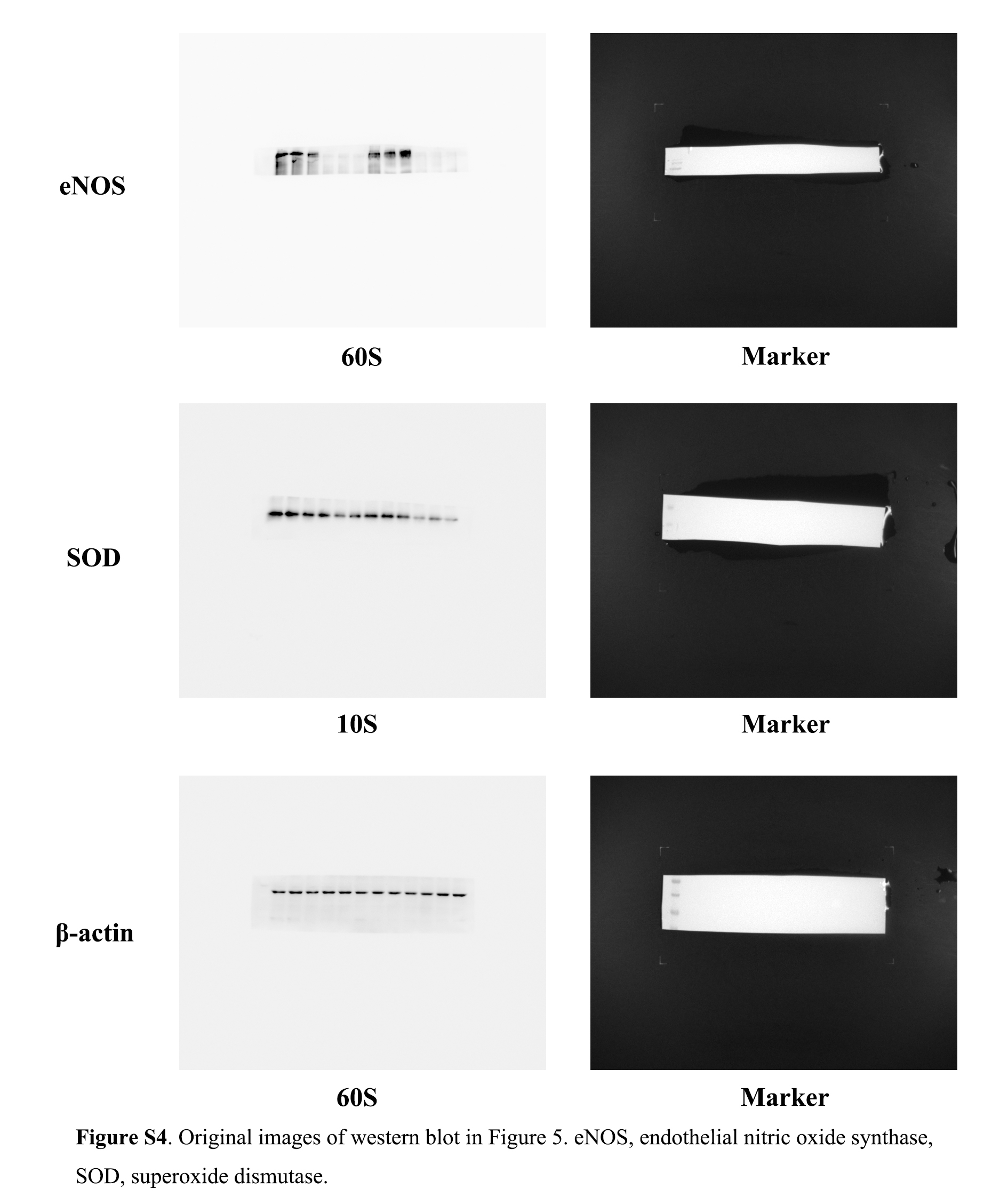

Supplement: Supplementary file 4 — Additional file 4: Figure S4. Original images of western blot in Fig. 5. eNOS, endothelial nitric oxide synthase SOD, superoxide dismutase. [file 12906_2022_3658_MOESM4_ESM.jpg]
